# Suppurative Flexor Tenosynovitis Secondary to Hematogenous Seeding

**Published:** 2017-06-01

**Authors:** Olivia Means, Mark Prevost, Rachel Seay, Ron Brooks

**Affiliations:** ^a^University of South Alabama College of Medicine, Mobile; ^b^University of South Alabama Medical Center Orthopeadic Residency Program, Mobile; ^c^Plastic and Reconstructive Surgery, Department of Surgery, University of South Alabama Medical Center, Mobile

**Keywords:** flexor tenosynovitis, hematogenous seeding, Kanavel, flexor sheath, hand infection

## DESCRIPTION

A 22-year-old woman was admitted for fever, intravenous drug use (IVDU), and bacteremia. After admission, she complained of knee and hand pain. Knee aspiration was sterile. She denied any injection into her left hand, and she had no puncture sites on examination. Kanavel's signs were present on examination, and she was taken urgently to the operating room for drainage. Her intraoperative cultures of methicillin-resistant *Staphylococcus aureus* (MRSA) matched her blood cultures taken upon admission, suggesting bacteremic seeding of the flexor tendon sheath.

## QUESTIONS

1. **What are the most frequent anatomic sites of hematogenous seeding due to bacteremia?**
2. **What is the anatomy of the flexor tendon sheath and how do you diagnose flexor tenosynovitis (FTS)?**
3. **What are the nonoperative and operative management strategies?**
4. **What is the postoperative management and expected outcome of FTS?**

## DISCUSSION

Bacteremia, defined as the presence of bacteria in the blood, may be transient, intermittent, or continuous. Specific host characteristics, such as an artificial device, a weak immune system secondary to medications or comorbid conditions, or risky behaviors such as IVDU, increase the likelihood of aggressive bacteremia, clinical infection, sepsis, or death.^1^ Common causative organisms include *S aureus*, β-hemolytic streptococci, *S epidermidis, S pneumoniae, Pseudomonas aeruginosa*, enterococci, gram-negative rods, yeast, or mixed organism infections.^1^^,^^2^ The most common locations of hematogenous seeding include the heart, brain, bones, and joints.^1^ The tendon sheath and bursa of the hand are closed spaces, and hematogenous seeding causing suppurative FTS is thus relatively rare. The vast majority of these cases are caused by a traumatic wound overlying the hand or flexor tendon sheaths.^2^^,^^3^

The tendon sheath and bursa of the hand exist in numerous anatomic variations. The closed space anatomy makes prompt diagnosis and treatment imperative to minimize the spread of infection and reduce subsequent stiffness, ischemia, necrosis, and amputation.^2^^-^5 A thorough history and physical examination are critical. Signs of penetrating injury should be sought as well as timing of the injury and other potential sources of infection, as in our case. White blood cell count, erythrocyte sedimentation rate, C-reactive protein all aid in diagnosis, but this remains a largely clinical diagnosis. Kanavel's signs help differentiate FTS from other hand infections: tenderness along the tendon sheath, finger held in flexion, pain on passive extension, and fusiform swelling.^2^^,^^6^

The treatment options are generally operative. Nonoperative management should only be attempted on patients who present within 48 hours of the trauma, are immunocompetent, and have no signs of abscess or necrosis. Management includes splinting, strict elevation, and intravenous antibiotics.^6^ Operative treatment includes both percutaneous and open irrigation and debridement. Percutaneous treatment may be effective in early infections, but open treatment is needed in most cases.^4^ Broad-spectrum antibiotics should be administrated regardless of the treatment decision and de-escalated once cultures are known. Most cases are a result of gram-positive organisms.^7^^,^^8^

Postoperative management varies, but often incisions are either left open or loosely closed. Some institute frequent soaks of the digit, and while others advocate for intermittent or continuous catheter irrigation for 24 to 48 hours.^4^ Care must be taken when irrigating so as not to cause a compartment-like syndrome. All patients should be initiated with early range of motion (ROM), beginning as soon as postoperative day 1. Outcomes are usually measured by final ROM, and studies show that both early treatment and those who received antibiotics have a much better final ROM.^4^ The dreaded need for an amputation in FTS was found to be 4.5%, and the greatest risk factors being diabetes, peripheral vascular disease, and renal failure; in addition, more than 40% of the patients needing an amputation in this particular study had a delay in treatment of at least 3 days.^8^

Our patient was initially admitted to the hospital for fevers and bacteremia due to IVDU. Blood cultures on admission were positive for MRSA. Consultation several days into her admission for knee pain (sterile aspiration) ultimately also led to the diagnosis of FTS. Kanavel's signs were all present for the middle/ring/index fingers, but there were no signs of trauma to the hand, and the patient denied any needlestick injuries to that hand. She underwent immediate drainage of the FTS of all 3 digits, but only the middle finger was purulent. The sheath was then irrigated with a 16-gauge angiocatheter in a proximal-to-distal direction until clear fluid was seen distally. Intraoperative cultures also revealed MRSA with matching sensitivities, consistent with hematogenous seeding of the sheath.

## Figures and Tables

**Figure 1:**
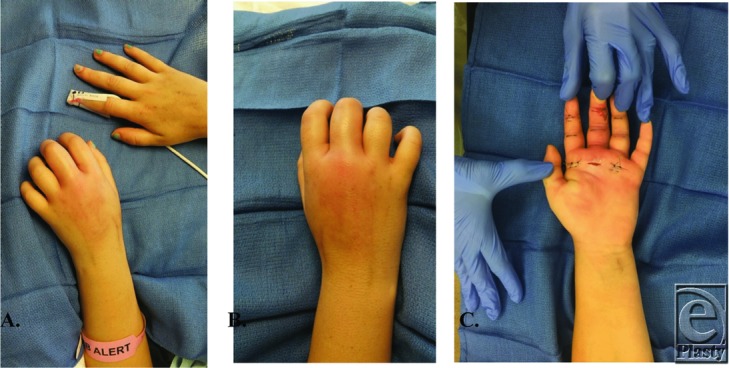
(a, b) The patient presented with localized pain to her left hand. The left hand was erythematous, taut, and tender to palpation. Specifically, her hand and fingers were held in a flexed position. She had pain on passive extension, fusiform swelling, and tenderness over the flexor tendon sheath of the middle/index/ring fingers. (c) Incision and drainage of the flexor tendon sheaths.
